# The effects of trypsin on rat brain astrocyte activation

**Published:** 2013

**Authors:** Masoud Fereidoni, Farzaneh Sabouni, Ali Moghimi, Shirin Hosseini

**Affiliations:** 1 Associate Professor, Department of Biology, Faculty of Sciences, Ferdowsi University of Mashhad, Mashhad, Iran; 2 Assistant Professor, National Institute of Genetic Engineering and Biotechnology (NIGEB), Tehran, Iran; 3 Professor, Department of Biology, Faculty of Sciences, Ferdowsi University of Mashhad, Mashhad, Iran; 4 Department of Biology, Faculty of Sciences, Ferdowsi University of Mashhad, Mashhad, Iran

**Keywords:** Astrocytes, Inflammation, Nitric Oxide, Trypsin

## Abstract

**Background:**

Astrocytes are cells within the central nervous system which are activated in a wide spectrum of infections, and autoimmune and neurodegenerative diseases. In pathologic states, they produce inflammatory cytokines, chemokines, and nitric oxide (NO), and sometimes they induce apoptosis. Their protease-activated receptors (PARs) can be activated by proteases, e.g. thrombin and trypsin, which are important in brain inflammation. The current study aimed to investigate the effects of different concentrations of trypsin (1 to 100U/ml) on cultured astrocytes.

**Methods:**

In the present study, two-day rat infants’ brains were isolated and homogenized after meninges removal, then cultivated in DMEM + 10% FBS medium. 10 days later, astrocytes were harvested and recultivated for more purification (up to 95%), using Immunocytochemistry method, in order to be employed for tests. They were affected by different concentrations of trypsin (1, 5, 10, 15, 20, 40, 60, 80, and 100 U/ml). To reveal the inflammation progress, NO concentrations (the Griess test) were assessed after 24 and 48 hours.

**Results:**

The results showed that trypsin concentration up to 20 U/ml caused a significant increase in NO, in a dose-dependent manner, on cultured astrocytes (P < 0.001). Trypsin 20 U/ml increased NO production fivefold the control group (P < 0.001). At higher concentrations than 20 U/ml, NO production diminished (P < 0.001). At 100 U/ml, NO production was less than the control group (P < 0.001).

**Conclusion:**

Inflammatory effects of trypsin 5-20 U/ml are probably due to the stimulation of astrocytes’ PAR-2 receptors and the increasing of the activation of NF-κB, PKC, MAPKs. Stimulation of astrocytes’ PAR-2 receptors causes an increase in iNOS activation which in turn leads to NO production. However, higher trypsin concentration possibly made astrocyte apoptosis; therefore, NO production diminished. These assumptions need to be further investigated.

## Introduction

Astrocytes, the stellar shape cells, are abundant cells within the central nervous system (CNS) with a population fivefold that of neurons.^1^ They could be activated and can exert different functions on neurons in pathologic circumstances like trauma, infection, hypoxia, ischemia, and brain injuries.^2^ Reactive alterations in the astrocytes including, hypertrophia, hyperplasia, and glial filaments (glial fibrillary acidic protein, GFAP), are accumulations within the cells. Moreover, activated astrocytes can produce a vast spectrum of neurotoxic mediators, including nitric oxide (NO), reactive oxygen species (ROS), and proinflammatory cytokines like tumor necrosis factor-α (TNF-α), and interleukin-1 and -6. These molecules directly act on neighboring cells and cause more activation in other astrocytes and microglial cells.^3^

The wide range of investigation in this respect show that long term activation of astrocytes is very important for neurotoxin production. Neurotoxin production is effective on the progress of neurodegenerative disease, as reactive astrocytes are more abundant in the brain of patients with neurodegenerative diseases such as Alzheimer's, Huntington's, and Parkinson's diseases.^4^ NO, is a small diffusible molecule, which is involved in a wide range of physiological and pathological activity in the CNS. A small amount of NO in the CNS is involved in neural development, morphogenesis, and plasticity, but excessive NO production within the CNS can cause neuronal damages during the brain ischemia and neuronal degeneration at different pathologic states.^5^ In regard to this concentration-dependent bi-functional action of the NO, regulation of NO level in the CNS is important. High NO level is produced after high-level expression of iNOS (inducible nitric oxide synthase) gene. Many studies have shown that iNOS gene expression is under the controlled regulation of NF-κB and MAPKs (mitogen-activated protein kinase).^6^ Besides NO, ROS, and proinflammatory cytokines, some serine proteases such as thrombin, tPA (tissue plasminogen activator),and trypsin, extracted from non-pancreatic tissues, also cause glial cell activation.^7, 8^ Recent studies have revealed the presence of trypsin and other proteases similar to trypsin, in the mammalian brain tissue.^9^ Trypsin is involved in a vast spectrum of cellular functions such as digestion, inflammation, immune responses, tumor metastasis, and nociception.^10^

Serine proteases are involved in inflammation and immune responses via activation of a family of G proteins coupling receptors (GPCRs) called protease-activated receptors (PARs). This family has four known members until now PAR-1 to -4, which present on the cell membrane of epithelial, endothelial, fibroblast, neuron, glial, and immune cells. PAR receptors play key roles in nervous system development, synaptic plasticity, neuro degeneration, NO dependent vasodilatation, cytokine production, and neural inflammation.^11, 12^ Serine proteases produce a proteolytic cleavage on the extracellular domain of their receptors, which raise a new N-terminal part on the receptors, and then run the signaling cascade. PAR-1, -3, and -4 are activated by thrombin, while PAR-2 can be activated by trypsin and tryptase.^13^ Studies have confirmed that PAR activation is accompanied with neurologic disorders initiation. Level of proteases such as thrombin, trypsin, tryptase, and specific neuronal proteases, including P22 and B-50/GAP-43 (SFRB60) within the CNS are increased following brain injuries and because of blood-brain barrier dysfunction. These enzymes, in turn, activate PARs and affect glial cell function by activation of the astrocytes, microglial, and inflammatory cells, which induce apoptosis, and then the degeneration of neighboring neuronal cells both in vivo and in vitro.^14^ In other circumstances, a disruption of balance between the proteases and their endogenous inhibitors (such as serpin) can cause the exhibition of inflammatory response in the brain. This is due to an elevation in PAR receptor expressions during brain injuries.^15^ Although the reports regarding the physiologic and pathologic roles of trypsin in the CNS are frequent, mechanism of its action on the CNS is not clear and is important to be investigated. The aim of this study is to perform in vitro investigation of trypsin action on the astrocytes derived from rat brain.

## Materials and Methods

One to two-day infant Wistar rats which were bred in the National Institute of Genetic Engineering and Biotechnology (NIGEB) of Tehran were used. Animals were housed under conditions of 12h light/12h dark, 22 ± 2 °C temperature, and ad libitum feeding conditions. All the experiments were running under the laboratory animal welfare and ethics rules.^16^

**Figure 1:**
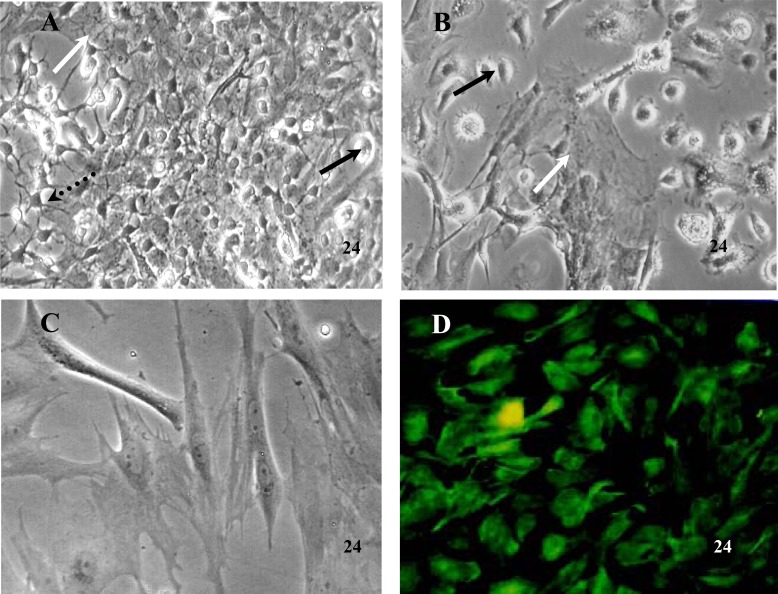
(A) Primary neural system mixed cell culture after 10 days; solid black arrow is showing microglia, solid white arrow is showing astrocytes, and dashed arrow is showing oligodendrocytes (32X by the phase contrast microscope). (B) Cell culture after trypsin incubation for cell dissociation; solid black arrow is showing microglia attached to the flask substrate, and solid white arrow is showing the dissociated astrocytic layer (32X by the phase contrast microscope). (C) Isolated and cultured astrocytes within the 24-well plate after 24 hours of incubation (32X by the phase contrast microscope). (D) Astrocyte special immunostaining using GFAP antibody for purified astrocyte culture (32X by the fluorescence microscope).

*Primary Mixed Cell Culture*- Using the culture method of McCarthy and de Vellis, primary cultures were prepared from the brain of one to two-day rat infants.^17^ The rat infants were decapitated under CO_2_ light anesthesia, then the brain was separated from the head very fast, after removing pia mater. Then, the brain was crashed by scalpel and cells were dissociated mechanically using pipettage method. The yielded cell suspension was cultured in T75 flasks containing DMEM (Dulbecco's modified Eagle's medium) with 10% FBS (fetal bovine serum). Cells were incubated in the 37 °C temperature, 5% CO_2_, and efficient humidity; the flask medium was renewed every two days until day 7th to 10th (Figure 1A).

*Astrocytes Culture Purification*- Flasks containing the primary mixed culture were shaken gently for 10 minutes. Based on the cells power to stick to the culture substrate, this produces microglial cells and oligodendrocytes on the medium surface disassociate from the sticky flask floor astrocytes. The supernatant was removed. Then, cells in the flasks were incubated with trypsin-EDTA for 5 minutes. Some microglial cells were sticking to the flask floor with more resistance to the action of trypsin to be disassociated from the floor. Therefore, astrocytes were dissociated from the floor, and microglial cells remained sticking to the floor (Figure 1B). Astrocytes were harvested and transferred to a 24-well plate; each well containing 10^5^ cells in volume of 500 µl DMEM culture medium containing 10% FBS. Before any treatment, cells in the plates were incubated for 24 hours to cover at least 80% of each well floor (Figure 1C). For determination of the astrocyte culture purification, astrocytic special immunocytochemical staining, GFAP antibody (Santa Cruz Biotechnology) as an astrocytic marker, was used.^18^

*Immunocytochemical Staining*- Isolated, purified, and cultured astrocytes within a 24-well plate, were fixed for 10 minutes by methanol after 24 hours of incubation. Then, in order to make astrocyte diffusible, 1 ml of 2% solution of Triton X100 was added to each well for 5 minutes. After 5 minutes, they were washed two times with PBS solution, and 1ml of 5% solution of BSA (bovine serum albumin) was added to each well. They were incubated at room temperature for 45 minutes. After removing the BSA solution, 200 µl of first antibody solution for GFAP by a dilution ratio of 1/200 was added to each well and they were incubated for 24 hours at 4°C over a shaker. In the next step, after removing primary antibody and cell washing by PBS solution, 100 µl of second antibody solution (polyclonal rabbit anti-mouse IgG/FITC, sigma) by a dilution ratio of 1/50 was added to each well. They were incubated for 2 hours over a shaker in the dark and at room temperature. Finally, after removing the second antibody solution and washing by PBS solution, cells were observed using fluorescent microscope (Figure 1D).

*Astrocyte Trypsin Treatment*- First, mediums over the cells on the 24-well plate were removed and renewed by DMEM/F12 medium containing 2% FBS. This is because more FBS leads to naturalization of the trypsin effects. Then wells were treated by different concentrations of trypsin (Sigma T4799) including 0, 1, 5, 10, 15, 20, 40, 60, 80, and 100 U/ml.

**Figure 2:**
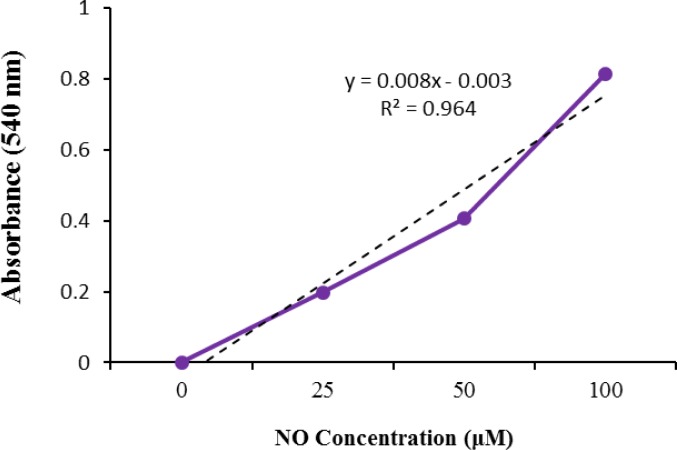
Standard curve for sodium nitrite: It shows the absorbance for the determined concentrations of 25, 50, and 100 µM of sodium nitrite. Calculation of NO level is possible by using the curve and mathematical function of the tangent line to the curve (y = 0.008x-0.003); y is the absorbance read by the microplate reader device and x is NO level with the unit of micro molar.

*Nitric Oxide Assessment*- 24 and 48 hours after astrocytes being treated by different concentrations of trypsin, the level of produced NO was assessed using the Griess method based on the assessment of the end product of nitrite. Griess method uses a colorimetric reaction for measuring the NO^2-^(nitrite) level in aqua's solution. Griess introduced this assessment based on diazotization for the first time.^19^To perform, 100 µl of Griess reagent is added to 100 µl of suspend solution over the cell cultures in each well, which are already centrifuged at 1000 rpm, then kept for 20 minutes in the dark and at room temperature. Then, absorbance is measured at the wave length of 540 nm using ELISA reader instrument (Microplate reader Labsystems Multiskan). Standard curve for sodium nitrite was used to acquire the level of NO which was produced by treated astrocytes; DMEM/F12 was used as blank (Figure 2).

*Statistical Methods*- Data are shown as mean ± SEM. Significance level for the effect of different trypsin concentrations was assessed using one way ANOVA and significant differences between the means were assessed by post-test of T_tukey_. P-values of less than 0.05 were considered as statistically significant. Statistics were performed by using the GraphPad Prism (version 5.0; GraphPad Software Inc., USA).

## Results

The results of astrocyte special immunocytochemical staining showed that final medium for cultures, which were derived from the brains of rat infants, contained astrocytes with a purity of almost 95% (Figure 1).

**Figure 3:**
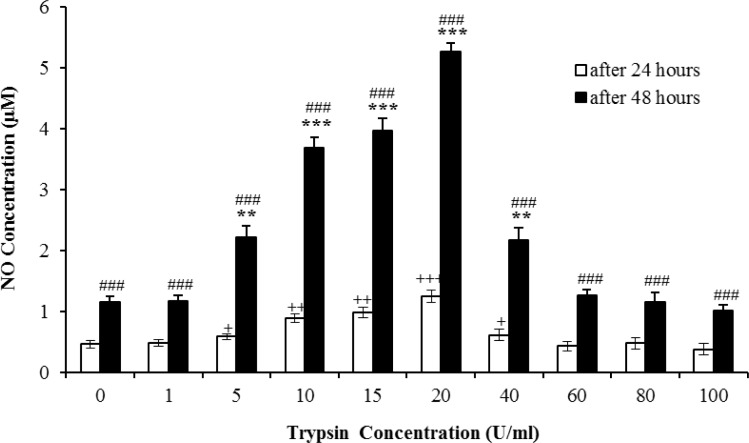
Comparison of NO level produced by astrocytes, which were treated by different trypsin concentrations from 0 to 100 U/ml after 24 and 48 hours using NO assessment method of Griess.

*Results of NO assessments*- The first pair of columns in Figure 3 which represent the control group (without trypsin treatment) show that there were no excessive nitric oxide production after 24 and 48 hours as NO was around the minimum level. However, NO production increased in a concentration dependent manner for the astrocytes which were treated by trypsin concentrations to 20 U/ml [F (11,96) = 187.97, P < 0.001]. At concentration of 20 U/ml, NO production was almost 5 times more than the same for the control group (P < 0.001). Part of Figure 3 which is shows the trypsin treating groups from 20 U/ml to 100 U/ml shows that more trypsin concentration attenuated the NO production by astrocytes, with a concentration dependent manner [F (9,81) = 169.83, P < 0.001]. Even for the trypsin treated astrocyte by concentration of 100 U/ml, the NO production was less than the cells in the controls which had no trypsin treatment (P < 0.001). Moreover, Figure 3 shows that 24 and 48 hours after trypsin treatment, NO production processes (concentration dependency) were similar. However, in contrast to 24 hours, NO production for all groups was augmented after 48 hours (P < 0.001).

Figure 3 shows that mean NO level produced by astrocytes, which are treated by different concentrations of trypsin (5 to 40 U/ml) increased significantly after 24 and 48 hours (***P < 0.001, *P < 0.01, + + +P < 0.001, + + P < 0.01, and +P < 0.05 in contrast to controls (the cells without trypsin treatment). NO production, in all groups, increased after 48 hours in respect to 24 hours (###P < 0.001). Data were presented as mean ± SEM, n is minimum repeats of cell cultures for each group (n = 9 for all groups).

## Discussion

Astrocytes are cell responsible for nourishment, immunity, protection, and CNS homeostasis. They are activated in pathologic circumstances and produce nitric oxide frequently.^2^ NO can exert neurotoxic effect on the neurons using different mechanisms, including enzymatic regulations, DNA degradation, oxidative reactions, and mitochondrial respiratory chain inhibition.^5, 20^ They also inhibit neuronal energy supply, antioxidants’ effects, and glutamate reabsorption, which leads to neuronal cell apoptosis.^20, 21^ Because astrocyte activation is accompanied with brain injuries, destroyed cells in the brain tissues or blood vessels release components, which activate astrocytes more and more.^4^ Many studies have shown that in diseases such as Alzheimer's disease, HIV induced encephalitis, and acute/chronic brain ischemia, expression of some serine proteases such as thrombin, trypsin, and tryptase can increase.^22, 23, 24^

These proteases activate or inhibit some substrate, one of which is PAR receptors in the brain. As mentioned in the introduction, each type of PAR receptor is activated by its special protease, for instance, thrombin activate PAR-1 while PAR-2 is cleft and activated by trypsin on the macrophages.^12, 25^ Recent findings revealed that trypsin is present in the CNS and plays a role in neuronal modulation.^26^ PAR-2 is also expressed on the smooth muscles, neurons, microglia, astrocytes, and C6 glioma cells. Activated PAR-2 exert different effects on tissues such as gastrointestinal muscle relaxation, nervous system inflammation, nociception signaling, and neuronal cell death.^27^ However, physiological and pathological roles of PAR-2 receptors in regard to trypsin actions and astrocyte activities remain to be elucidated. The acquired results of the present study show that astrocytes can change NO production by being exposed to different concentrations of trypsin (Figure 3). There are three possible mechanisms that can be suggested for activation and inflammatory effects of trypsin on astrocytes, which are explained below. However, each one needs to be proven experimentally in the presence of trypsin and astrocytes.

1. *Increase in iNOS gene expression*- Recent studies have shown that in C6 glioma cells and microglial cells, PAR-2 activation under the effects of proteases (such as trypsin and thrombin) led to an increase in iNOS gene expression and NO production.^28, 29^Meli et al. showed that PAR activation via thrombin in C6 glioma cells led to an increase in iNOS gene expression followed by NO production.^30^ It was shown that PAR-2 stimulation in microglial cells can lead to iNOS gene expression and increase in NO production, and TFLLR peptide, which is PAR-1 agonist, TFRGAP peptide, which is PAR-3 agonist, and GYPGKF peptide, which is PAR-4 agonist, are able to increase the NO production potency of rat brain microglia.^29^
2. *Activation of signaling pathways within the cell, including PKC and MAPKs*- Recent investigations have shown that thrombin leads to increase in the intracellular Ca^2 +^ level by stimulating the PAR-2 in the murine astrocytes and C6 glioma cells in a dose/concentration dependent manner. Ca^2 +^signaling pathway requires special cellular switches such as PKC, which turn on the transmission of Ca^2 +^ signal to the cell responses.^28, 31^ PKC activation can induce iNOS gene expression elevation, which in turn leads to increase in NO production. It is reported that PKC inhibition of the cells, which previously had been excited by thrombin, using Go6976 has led to inhibit the NO production, while PKC activators (like PMA) acted in the same way as thrombin.^29^ These finding show that NO production is probably passing through the PKC signaling pathway after thrombin action on PAR-2 which existed on the astrocytes, C6 glioma cells, and microglial cells. Furthermore, many signals activate MAPKs. This is also reported for activated PAR receptors; when thrombin activates PAR receptors and thus elevates iNOS gene expression and NO production by microglial cells, inhibition of the MAPKs such as ERK, JNK, and P38 can lead to diminishing NO production.^32^
3. *NF-κB activation*- The iNOS gene's level of transcription is regulated. An investigation on the microglia and astrocytes has shown that transcription factor of NF-κB has a special site in the iNOS gene promoter. Astrocyte activators such as β-amyloid, γ-interferon, and gangliosides can activate NF-κB and increase gene expression of iNOS, and so increase astrocyte NO production.^33, 34^ It has been reported that PAR activation by proteases, like thrombin and trypsin, on the microglial cells, astrocytes, and C6 glioma cells activates NF-κB and increases NO production. PDTC induced NF-κB inhibition leads to diminishing NO production.^29^

One of the most important questions which remain to be answered is “what can lead the physiological roles of trypsin and related proteases toward pathologic states within the brain?”. We do not have a determined concentration for proteases in the brain in the pathologic or injured states. However, it is known that neurons can produce such proteases which can be released by regulated releasing pathways, such as the exocytosis of dense core granules in the nerve axonal terminals and even potential releasing at dendrite regions. These neuronal proteases like trypsin, mesotrypsin, P22 and, neurosin, in turn, can activate PARs on neurons and glial cells.^35^

## Conclusion

In conclusion, the present findings suggest that trypsin concentrations up to 20 U/ml can activate astrocytes and increase astrocyte NO production. This may be followed by PAR receptor activation of astrocytes and iNOS gene expression through PKC, MAPKs, and NF-κB signaling pathways which need to be investigated more accurately. However, the present results also show that trypsin, at higher concentrations, leads to alleviation in NO production by astrocytes. This may be because of apoptosis induction after potent astrocytes’ PAR receptor stimulation that leads to diminishing of NO level in the culture medium. This assumption also needs to be further investigated.
